# Colonization Resistance of Symbionts in Their Insect Hosts

**DOI:** 10.3390/insects14070594

**Published:** 2023-06-30

**Authors:** Zhengyan Wang, Hanzi Yong, Shan Zhang, Zhiyuan Liu, Yaru Zhao

**Affiliations:** 1 School of Food and Strategic Reserves, Henan University of Technology, Zhengzhou 450001, China; 2 School of Grain Science and Technology, Jiangsu University of Science and Technology, Zhenjiang 212100, China

**Keywords:** insect, defensive symbiont, exogenous pathogen, colonization resistance, coevolution

## Abstract

**Simple Summary:**

Insects live in an incredibly complex environment and are constantly exposed to different microbiota, and some of them are beneficial while some are harmful to hosts. Colonization of some beneficial symbionts can increase host resistance against exogenous pathogens. This review summarizes the mechanisms by which symbionts contribute to the host’s immune capacity. Adaptations of symbionts and their insect hosts to maintain such symbiotic relationships, and the significance of such relationships in the coevolution of symbiotic systems are also discussed to provide insights into the in-depth study of the contribution of symbionts to host physiology and behavior.

**Abstract:**

The symbiotic microbiome is critical in promoting insect resistance against colonization by exogenous microorganisms. The mechanisms by which symbionts contribute to the host’s immune capacity is referred to as colonization resistance. Symbionts can protect insects from exogenous pathogens through a variety of mechanisms, including upregulating the expression of host immune-related genes, producing antimicrobial substances, and competitively excluding pathogens. Concordantly, insects have evolved fine-tuned regulatory mechanisms to avoid overactive immune responses against symbionts or specialized cells to harbor symbionts. Alternatively, some symbionts have evolved special adaptations, such as the formation of biofilms to increase their tolerance to host immune responses. Here, we provide a review of the mechanisms about colonization resistance of symbionts in their insect hosts. Adaptations of symbionts and their insect hosts that may maintain such symbiotic relationships, and the significance of such relationships in the coevolution of symbiotic systems are also discussed to provide insights into the in-depth study of the contribution of symbionts to host physiology and behavior.

## 1. Introduction

Multicellular organisms are continuously exposed to a variety of microorganisms, including bacteria, fungi, protozoa, or viruses. Among them, microorganisms that are potentially harmful to multicellular organisms are called exogenous pathogens. Multicellular organisms have developed cellular and molecular defense mechanisms to protect themselves from exogenous pathogens, which is collectively called immunity [1]. In vertebrate hosts, there is a two-tiered immune defense strategy: innate immunity and adaptive immunity. During the first invasion of an exogenous pathogen into multicellular organisms, basic immune mechanisms such as recognition of pathogen-associated molecular patterns (PAMPs) via host pattern-recognition receptors (PRRs), expression of antimicrobial peptides (AMPs), and activation of phagocytic cells participate in the elimination of pathogens. These basic immune mechanisms are generally termed the innate immune system. When the same pathogen invades the vertebrate host for the second time, specific antigen recognition receptors on the lymphocytes could recognize the pathogen and activate a more specific defense response, i.e., adaptive immunity, thereby increasing the efficacy of host immune responses [2]. In contrast, insects lack an adaptive immune system and thus rely on a powerful innate immune system that fights against pathogen infections [3].

Although immune responses vary significantly within insect taxa [4], the innate immune system of insects generally encompasses physical barriers, humoral responses, and cellular responses [5]. The peritrophic matrix (PM) is a non-cellular film comprising chitin and glycoproteins. The PM functions as a physical barrier to prevent the midgut epithelium being destroyed by abrasive food particles, digestive enzymes, and pathogen infection through food ingestion [6]. Although the PM acts as a selectively permeable molecular sieve, it does not protect hosts against all pathogens [7]. Cellular and humoral immune responses are activated when pathogens pass through the PM and enter the hemolymph. The cellular immunity performed by hemocytes involves phagocytosis, nodulation, and encapsulation [8]. The humoral defenses comprise soluble effector molecules, including AMPs, reactive oxygen species (ROS), and products produced by proteolytic cascades such as the phenoloxidase (PO) pathway, which immobilize or eliminate pathogens in the hemolymph [9]. Cellular immunity and humoral immunity synergistically defend against pathogens and maintain microbial homeostasis within insects [10].

Symbionts play crucial roles in insect physiology and behavior, including food digestion, host nutrition, natural enemy defense, insecticide resistance, and mating preference [11,12]. In addition, some studies have shown that symbionts can upregulate host immune responses or inhibit the proliferation of pathogens through the production of antimicrobial substances or interspecific ecological competition, thereby protecting hosts against pathogens [13]. The mechanisms by which symbionts contribute to the host’s immune capacity is referred to as “colonization resistance” [14]. Accordingly, “defensive symbionts” are defined as conditionally beneficial microbes associated with hosts, including bacteria, archaea, fungi, viruses, and members of the microbiome when hosts are challenged with pathogens [15]. For example, infection with the endosymbiont *Wolbachia* confers resistance of *Drosophila melanogaster* to the *Drosophila* C virus and reduces the viral load in infected flies [16].

Here, we provide a review of the mechanisms about colonization resistance of defensive symbionts in their insect hosts. Adaptations of symbionts and their insect hosts to maintain such symbiotic relationships, and the significance of such relationships in the coevolution of symbiotic systems are also discussed to provide insights into the in-depth study of the contribution of symbionts to host physiology and behavior.

## 2. Mechanisms of Colonization Resistance

Defensive symbionts protect hosts against exogenous pathogens through three general mechanisms: (1) symbionts upregulate the expression of host immune-related genes and thereby enhance host resistance against exogenous pathogens; (2) symbionts produce antimicrobial substances and thus contribute to host defensive chemistry; and (3) symbionts provide protection to hosts by competitively excluding exogenous pathogens [17].

### 2.1. Upregulation of the Expression of Host Immune-Related Genes

Symbionts can help hosts defend against pathogenic infestations by upregulating the biosynthesis of host humoral immune effectors. In larval *D. melanogaster*, the occurrence of the naturally occurring avirulent *w*Mel strain of *Wolbachia* upregulates the expression of multiple genes linked to host humoral immune responses, including two AMP genes and a positive modulator for the immune deficiency (IMD) signaling pathway [18]. Likewise, gut symbionts of *Apis mellifera* [19], *Anopheles stephensi* [20], and the bean bug *Riptortus pedestris* [21] induce upregulation of the expression of immune effectors such as c-type lectins (CTLs), CLIP serine proteases, and AMPs and thus protect hosts from pathogens.

Some bacterial symbionts can upregulate the expression of host cellular immune-related genes. In the tsetse fly *Glossina morsitans morsitans*, expressions of prophenoloxidase and thioester-containing proteins (*tep2* and *tep4*) associated with cellular immune processes such as phagocytosis and encapsulation are downregulated, and phagocytosis, hemolymph coagulation, and melanin deposition are decreased dramatically in sterile individuals. Consequently, these flies are highly susceptible to hemocoelic infections with usually non-pathogenic *Escherichia coli* [22]. The susceptible immune phenotype demonstrated by sterile flies could be reversed by transplanting hemocytes from wild-type donor flies before infection. Additionally, when the mothers of intrauterine sterile tsetse larvae are fed with a diet added with *Wigglesworthia* cell extracts, the immune system of the larvae develops normally [23]. These results indicate that substances produced by *Wigglesworthia* play a critical role in the development and activation of the host immune system.

It is also found that the presence of symbionts could positively influence the generation and differentiation of host immune-related hemocytes. Compared with the wild-type individuals, sterile tsetse flies possess a lowered number of sessile and circulating hemocytes. This phenomenon may be attributed to a drastic decrease in the expression of two transcription factors, *lozenge* and *serpent*, associated with hematopoiesis. During embryogenesis in the closely related *Drosophila*, early hematopoiesis is differentiated based on the expression of *serpent*. Subsequently, *lozenge* guides the differentiation of *serpent*-expressing precursor cells into a specific lineage of hemocytes known as crystal cells [22]. The obligate intracellular symbiont *Wigglesworthia* upregulates the expression of the odorant binding protein 6 (obp6) in the gut of intrauterine tsetse larvae. The obp6 can induce systemic expression of *lozenge* and subsequent production of crystal cells (a type of hemocyte), which actuates the melanotic immune response in tsetse adults [24].

### 2.2. Symbiont-Mediated Insect Immune Priming

Until recently, it was believed that insects were deficient in immune memory as they possess no vertebrate-like specialized memory cells. Nevertheless, many studies have discovered immune memory-like responses in insects, i.e., immune priming, by which prior sublethal infestation with a pathogen enhances the host’s immune capacity to defend against subsequent infestation by the same pathogens [25]. Interestingly, some studies have found that the symbiotic microbiota is necessary for priming against pathogens in some insects. For example, *Tribolium castaneum* larvae with a significantly lower microbial load exhibit decreased survival upon secondary challenge with *Bacillus thuringiensis* spores when compared with larvae that are allowed to regain their microbiota prior to priming [26].

Mechanisms of insect immune priming elicited by symbionts have been demonstrated in the mosquito *Anopheles gambiae*. When the mosquito is rechallenged with the same *Plasmodium* parasite, elimination of the gut symbionts prevents elicitation of the priming response. The immune priming of the mosquito to *Plasmodium* includes a continuous increment in the expression of Evokin (a lipid carrier of the lipocalin family) and in its capability to transform arachidonic acid into lipoxins (a hemocyte differentiation factor). Invasion of *Plasmodium* ookinete to the midgut activates immune priming by eliciting the release of the mosquito lipoxin/lipocalin complex [27]. This process is triggered by the gut symbionts that enter the hemocoel and stimulate the differentiation of prohemocytes into granulocytes (a type of immune cell) when the *Plasmodium* ookinetes break the barrier between the gut and hemocoel [28].

### 2.3. Production of Antimicrobial Substances

Many studies have demonstrated that external symbionts could produce antimicrobial substances that help hosts defend against pathogens. Due to their eminent abilities to exploit a wide range of carbon and nitrogen sources and their extensive repertoire of secondary metabolites, different actinobacteria have been reported to protect ants, beetles, and wasps against pathogens by producing antimicrobial substances [29]. In the European beewolf *Philanthus triangulum*, the heritable symbiont *Streptomyces* secretes antibiotics that protect host cocoons from pathogen attacks during diapause in the soil [30]. *Actinobacteria* in the cuticle of the leaf-cutting ant *Acromyrmex subterraneus subterraneus* are capable of secreting antimicrobial substances to protect the host from *Metarhizium anisopliae* [31]. Some non-actinobacteria are also reported to produce antimicrobial substances. The symbiont *Burkholderia gladioli* dwelling on the egg surface of Lagriinae beetles produces an antibiotic cocktail containing toxoflavin, caryoynencin, macrolide lagriene, and isothiocyanate sinapigladioside to protect vulnerable eggs against virulent microbiota [32].

Some internal symbionts (extracellular and intracellular) can also produce antimicrobial substances to enhance host resistance to pathogens, among which proteobacteria are frequently reported. The gut symbiont *Pantoea agglomerans* produces phenols to inhibit the proliferation of *M. anisopliae* in the gut of *Schistocerca gregaria* [33]. *Serratia* AS1 has been genetically modified to secrete anti-*Plasmodium* effector proteins. Recombinant *Serratia* stably colonize the mosquito intestine, the female ovary, and male accessory glands and inhibit the proliferation of *Plasmodium falciparum* in mosquitoes [34]. Notably, some intracellular symbionts can also boost host immunity through the production of antimicrobial substances. The facultative endosymbiont *Regiella insecticola* in the hemolymph of the pea aphid *Acyrthosiphon pisum* inhibits the proliferation of the Entomophthorales fungus *Pandora neoaphidis* in the body cavity [35].

Regardless of whether endosymbionts modulate the host’s immune capacity by producing antimicrobial substances or by modulating the expression of host immune-related genes, there are multiple spatial barriers between endosymbionts and extracellular pathogens, and the signaling pathways by which pathogens trigger the immune response of endosymbionts are not well understood. Some studies have shown that there are receptors on the cell membrane of endosymbionts that can bind to viral capsid proteins [36], which at least indicates that information exchange between endosymbionts and viruses occurs.

### 2.4. Competitive Exclusion of Pathogens

In many cases, the beneficial microbiota can even be considered as an extension of the host immune system through competitive exclusion of pathogens [37]. Gut symbionts may defend the host by modifying gut physiological conditions, such as modulating the environmental pHs. Among the endemic symbiont groups frequently found within the *A. mellifera* microbiota, acetic acid bacteria (AAB) are acid-tolerant and capable of acidifying the pH during their growth. Changes in environmental pHs can likely affect the growth of acid-sensitive pathogenic bacteria that share with AAB symbionts the same gut micro-niche [13].

The abundance of the symbiont community corresponds with host defense capabilities against pathogens. The load and diversity of the midgut microbiota are positively correlated with the tolerance of *Spodoptera exigua* to *B. thuringiensis* [38] and the tolerance of *S. gregaria* to *Serratia marcescens* [39]. Maybe it is because the gut microbiota with high abundance exhibits greater potential to compete for ecological niches and nutrients with exogenous pathogens and to maintain the stability of the gut ecosystem. It has been found in humans that the resident gut microbiota could maintain or enhance the function of the gut mucosal barrier by competing with pathogens for adhesion sites, receptors, and nutrients [40]. Further in situ monitoring of the microbial distribution will help to reveal the antagonistic relationship between symbiotic and pathogenic bacteria within insects.

## 3. Balance of Host Immune Response

Gut symbionts have positive impacts on many aspects of host physiology and behavior. At the same time, the persistent presence of microbiota within insects poses a constant challenge to the host immune system [41]. Establishing a balance between excessive symbiont colonization and persistent stimulation of the host immune system is crucial to maintain such symbiotic associations. The former could be lethal to the host, while the latter may lead to symbiotic damage and host fitness cost [42]. As a result, the host immune system has developed different strategies to preserve its capability to respond to pathogens, while keeping beneficial symbionts alive and metabolically active [43]. These strategies include (1) degeneration of the host immune system [44]; (2) fine-tuned regulation of immune responses by host negative regulators [45]; and (3) compartmentalization of the symbionts in specialized symbiotic organs [46].

### 3.1. Degeneration of the Host Immune System

Immunologically, aphids are unusual when compared with many previously identified insects as they rely heavily on symbiotic bacteria for survival while are also frequently challenged with different microbial and multicellular parasites. The primary symbiont *Buchnera aphidicola* is compartmentalized into specialized cells called bacteriocytes [47], while facultative symbionts occur in both hemolymph and bacteriocytes, where they provide multiple benefits, including defenses against fungi and parasitoids [44]. The aphid genome lacks many predicted immunity genes, suggesting that its immune system is not as strong as that of other insects. Immune gene loss could reflect either relaxed selection to maintain costly immune functions as facultative symbionts [48] or compounds from host plants [49] can execute similar defensive functions, or an adaptation that has facilitated symbiotic bacteria to colonize the host. In contrast to such extreme host immune adaptations, it is more universal for the host to discriminate between symbionts and exogenous pathogens via fine-tuned immune responses.

### 3.2. Fine-Tuned Regulation of Immune Responses by Negative Regulators

The fundamental immune mechanisms of the insect gut, including the IMD signaling pathway and the dual oxidase-reactive oxygen species (DUOX-ROS) system, participate in the maintenance of microbial homeostasis [50]. Recognition of microbe-associated molecular patterns (MAMPs) by host PRRs triggers the IMD signaling pathway or the DUOX-ROS and subsequent immune responses. To date, only diaminopimelic acid-type peptidoglycan (DAP-PGN) and bacterial-derived uracil have been demonstrated to initiate the IMD signaling pathway and the DUOX-ROS, respectively [51].

If symbionts or pathogens could evolve species- or strain-specific MAMPs, the host immune system could easily distinguish between the symbionts and pathogens and decide whether to activate the immune response. For example, uracil is produced by the pathogen *Erwinia carotovora* subspecies *carotovora* 15 (Ecc15) but not by most of the symbionts in the gut [52]. The host can recognize the uracil and activate the DUOX-ROS, which facilitates host resistance to pathogen infections and maintenance of microbial homeostasis [53].

Unfortunately, MAMPs, such as peptidoglycans (PGNs), lipoplysaccharides (LPSs), ester phosphopeptides, glucans, and mannans, are greatly similar among the many different microbiota and could not be used to discriminate between symbionts and pathogens by the host immune system [54]. For example, DAP-PGN, which is commonly present in the membranes of Gram-negative bacteria and some Gram-positive bacteria like *Bacillus*, is recognized by PGN recognition proteins (PGRPs) and subsequently triggers the activation of the IMD signaling pathway for AMP production [51]. However, DAP-PGN is not a pathogen-specific molecular pattern as it usually occurs in both symbionts like *Acetobacter* and pathogens like Ecc15. Since these two bacteria can initiate the IMD signaling pathway, this pathway is not enough to elaborate how epithelial cells differentiate symbionts from pathogens [52].

**Figure 1:**
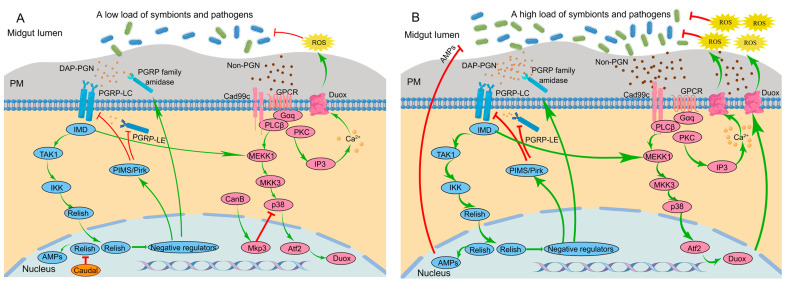
Signaling network of the insect immune system to modulate AMP and ROS production in the midgut of *Drosophila melanogaster* in the presence of a low microbial load (**A**) and a high microbial load (**B**). Red lines and green lines indicate active pathways, where the green lines indicate activation and the red lines indicate inhibition. The thickness of the line indicates the strength of the signal. AMPs, antimicrobial peptides; Atf2, activating transcription factor 2; CanB, calcineurin B; Cad99c, cadherin 99c; DAP-PGN, diaminopimelic acid-type peptidoglycan; Duox, dual oxidase; Gαq, G protein αq subunit; GPCR, G-protein-coupled receptor; IKK, IκB kinase; IMD, immune deficiency; IP3, inositol 1,4,5-trisphosphate; MEKK1, MAPK and ERK kinase 1; MKK3, MAPK kinase 3; Mkp3, MAP kinase phosphatase-3; p38, p38 mitogen-activated protein kinase pathway; PGRPs, peptidoglycan recognition proteins; PIMS/Pirk, PGRP-LC-interacting inhibitors of IMD signaling; PKC, protein kinase C; PLCβ, phospholipase C-β; PM, peritrophic matrix; ROS, reactive oxygen species; TAK1, transforming growth factor-β-activated kinase 1.

Insects have evolved a signaling network to regulate microbial loads via negative regulators [55]. The insect immune signaling network that controls the production of AMP and ROS in the midgut of *D. melanogaster* is often used as a model to illustrate the regulation of the immune response by negative regulators (Figure 1).

#### 3.2.1. IMD-AMP Pathway

The IMD signaling pathway possesses two PGRPs, namely the transmembrane protein receptor PGRP-LC and the cytoplasmic receptor PGRP-LE [56]. These receptors recognize DAP-PGN and activate the downstream transcription factor Relish, which subsequently translocates into the nucleus and modulates AMP gene expression [51].

In the presence of a low load of symbionts, low amounts of PGNs released from the symbionts bind to the PGRPs, which induces the basal activity of the IMD signaling pathway. Usually, three kinds of negative regulators work together to inhibit excessive immune responses of the host: the PGRP family, PGRP-LC-interacting inhibitors of IMD signaling (PIMS, also called Pirk), and Caudal. The PGRP family with amidase activity, such as PGRP-LB, PGRP-SB1, PGRP-SB2, PGRP-SC1a, PGRP-SC1b, and PGRP-SC2, are produced by epithelial cells in the midgut and degrade the pro-inflammatory PGNs into nonactive forms. This helps to maintain a low level of resident microbiota-derived PGNs [56]. The protein PIMS inhibits the activity of PGRP-LE and PGRP-LC [57]. The homeobox transcription factor Caudal exerts specific suppression on AMP gene transcription in epithelial cells by binding to promoter regions [58] (Figure 1A).

When the bacterial load is high, for example, in the presence of pathogens, enhanced levels of PGNs results in robust activation of the IMD signaling pathway. This triggers mass translocation of Relish into the nucleus, thereby overcoming Caudal repression and inducing AMP transcription [14] (Figure 1B).

#### 3.2.2. DUOX-ROS

DUOX takes charge of microbicidal ROS generation in response to a broad range of stimuli [59]. Unlike the gut IMD signaling pathway, it is the uracil, not PGN, that acts as an agonist to induce DUOX-dependent ROS generation [55]. The DUOX-dependent ROS generation in insect guts is primarily modulated by two signaling pathways. One pathway, known as the “DUOX-activity pathway”, is triggered by the Cad99C/PLCβ/PKC-dependent signaling endosome, which subsequently induces intracellular Ca^2+^ release (i.e., calcium signaling) to modulate the enzymatic activity of DUOX. The other pathway, known as the “DUOX-expression pathway”, modulates DUOX expression through sequential activation of the MEKK1-MKK3-p38-Atf2 pathway [60]. The initiation of both pathways is essential for mass generation of ROS. Notably, while the DUOX-expression pathway can be triggered by PGNs, the DUOX-activity pathway cannot [61]. Nevertheless, initiation of the DUOX-activity pathway is essential for generating DUOX-dependent ROS. Therefore, bacterial-derived PGNs alone are insufficient to initiate DUOX-dependent ROS generation [62] (Figure 1).

The bacterial-derived uracil serves as a non-PGN ligand for DUOX-dependent ROS production [55]. The uracil is recognized via a G-protein-coupled receptor (GPCR). DUOX-activity pathway-dependent signaling endosomes facilitate the signal amplification by eliciting phospholipase Cβ (PLCβ)-dependent Ca^2+^ mobilization to produce ROS. The signaling endosomes are then degraded to avoid excess ROS production [63] (Figure 1). It is worth noting that uracil is not present in gut resident symbionts, permitting peaceful colonization without DUOX activation, while uracil released from occasional pathogens triggers persistent inflammation [53].

In the presence of a low load of symbionts and pathogens, recognition of uracil by GPCR rapidly stimulates PLCβ via Gαq, which subsequently mobilizes intracellular Ca^2+^ through the production of inositol 1,4,5-trisphosphate (IP3) to produce ROS. PGNs from symbionts can activate the DUOX-expression pathway [62]. However, p38 activation is suppressed by the dual phosphatase MKP3, which is sequentially caused by calcineurin B (CanB) [14]. Inactivation of p38 due to weak PGN-dependent and non-PGN-dependent signals prevents excessive stimulation of the DUOX-expression pathway (Figure 1A). The basal activity of the DUOX system, mediated by calcium signaling, is enough to maintain the homeostasis of the symbiotic community.

When the bacterial load increases, gut epithelial cells are confronted with serious bacterial infections that cannot be effectively eliminated by the basal activity of the DUOX system through calcium signaling alone. Therefore, positive regulation of the DUOX expression pathway is activated [61]. DUOX expression is promoted through p38 signaling activated by both strong PGN-dependent and non-PGN-dependent signals. Additionally, DUOX activity is enhanced by further calcium mobilization [14] (Figure 1B).

### 3.3. Compartmentalization of Symbionts

An insect host possesses many habitats available to symbionts, and the fitness of the symbionts and the insect host can be dictated by the location of the symbionts in the host. A microbe may be beneficial in some parasitized tissues, but it may be detrimental to the host or fail to persist in other parasitized tissues. In the tobacco hornworm *Manduca sexta*, *Enterococcus faecalis* undergoes a transition from the beneficial gut symbiont to the hemocoelic pathogen after translocating from the gut to the hemocoel due to extensive melanization induced by *E. faecalis* [64]. The compartmentalization strategy isolates the symbionts in specialized tissues, providing an optimal environment for their metabolic activities and bringing them under control while avoiding excessive proliferation and virulence.

For many symbiotic associations, the favored residence for symbionts is a specialized organ (or part of an organ) that functions to maintain the conditions and resources available to the symbionts and to ensure that hosts exploit the benefits from the symbiont services [65]. The midgut of the bean bug *R. pedestris* is separated into four distinct regions, namely M1, M2, M3, and M4B, and the symbiotic region of M4. The M4 region possesses two lines of crypts that are colonized by a high load of the symbiont *Burkholderia*. To maintain the presence of the immune-susceptible *Burkholderia*, *R. pedestris* suppresses immune responses in the M4 region [21]. Endosymbiont seclusion within bacteriocytes that is usually organized in dedicated organs, often called bacteriomes, has been found in many insects, such as aphids [66], planthoppers [67], cicadas [68], and beetles [69]. The compartmentalization plays a crucial role in shielding them from host immune responses while evading persistent host immune stimulation [41].

Although endosymbionts that are segregated within the bacteriocytes are less affected by the host humoral immunity, the host still develops adapted local immune responses to maintain symbiont homeostasis. Tsetse PGRP-LB can decompose PGNs originating from *Wigglesworthia* and can inhibit the actuation of symbiont-damaging host immune defenses. Tsetse *pgrp-lb* is expressed in the *Wigglesworthia*-harboring midgut region, and its expression level is positively related to the symbiont load. Tsetse flies free from *Wigglesworthia* possess significantly less PGRP-LB than control adults. RNA interference-mediated removal of PGRP-LB triggers the IMD signaling pathway and induces AMP synthesis, which reduces the *Wigglesworthia* load [70]. In the maize weevil *Sitophilus zeamais*, coleoptericin A (ColA), the only constitutively expressed AMP in the bacteriocytes, specifically targets endosymbionts and suppresses their cytokinesis, thereby inhibiting the division and dispersion of bacterial cells throughout insect tissues [71].

## 4. Adaptation of Symbionts to Host Immune

Biofilms have been defined as a film of microbial communities enclosed by a matrix of extracellular polymeric substance [72]. Molecular mechanisms of the formation and role of biofilms are fully explained in pathogens. Many pathogenic microbes, such as *E. coli* [73], *Salmonella Typhimurium* [74], *Acinetobacter baumannii* [75], and *M. anisopliae* [76], avoid host immune responses by masking themselves with protective biofilms. When dwelling in a biofilm, the bacteria are surrounded by extracellular polymeric substances, and PAMPs are less exposed to the immune system [77]. For example, a collagenous protective coat (MCL1) enables *M. anisopliae* to avoid being recognized and attacked by hemocytes in *M. sexta*. MCL1 conceals antigenic structural components of the cell wall such as β-glucans, and its hydrophilic negatively charged character repels hemocytes [76]. The biofilm could also defend pathogens against host immune responses by cloaking them from AMPs [78].

In contrast, the association between biofilms and symbiont colonization within insects remains poorly understood. Polymorphisms in the exposed loop domains of the main bacterial surface protein, outer-membrane protein A (OmpA), exemplify a microbial evolution that modulates host tolerance to symbionts. OmpA participates in the biofilm formation of bacteria, such as *E. coli* and *Sodalis glossinidius* [79]. A sequence analysis of the *Sodalis* OmpA gene reveals that exterior loop domains of the OmpA protein have amino acid insertions and/or substitutions in comparison with that of *E*. *coli* and other closely related exogenous and pathogenic bacteria [80,81]. Differences in the phenotype of *Sodalis* and *E. coli* OmpA are consistent with the infection results, as tsetse flies could tolerate thoracic superinfection by *Sodalis* and *E. coli* mutants (expressing *Sodalis* OmpA) but are highly sensitive to infection with the wild-type *E. coli* [79]. As OmpA is also considered a PAMP [82], recognition of OmpA mutations in avirulent *Sodalis* induces strong host immune responses, while virulent *E. coli* does not. This indicates that this protein plays a vital role in the successful symbiosis of *Sodalis* and its host.

The gut microbiota of the mosquitoes *Aedes aegypti* and *Culex pipiens pallens* triggers the host expression of CTLs, which cover the bacterial surface and resist AMP activity. Several soluble CTLs coat bacterial surfaces via polysaccharides, which facilitates establishment of a variety of bacterial strains or maintenance of the gut microbial community in mosquitoes. The CTLs coat could not only exclude AMPs from contacting symbiotic bacteria but also mask the MAMPs from being recognized by PRRs of the gut epithelial cells. Furthermore, genetic manipulation of CTLs fails to change the expression of AMPs and many other important immune genes, suggesting that CTLs do not intervene with PRR-mediated signaling. The release of CTLs is an immunoregulatory strategy to defend symbionts against constitutive elimination modulated by AMPs, allowing co-adaptation of the gut microbiota in mosquitoes [83].

## 5. Coevolution of Defensive Symbiosis

In a mutualistic symbiosis, the symbiont evolves mechanisms to break the physical, cellular, and immune barriers presented by the host in order to colonize host cells and to achieve transmission to offspring. In the defensive symbiosis, the symbiont has developed mechanisms to defend the host against pathogens while protecting themselves from being destroyed by the host immune responses. On the other hand, the host modulates its immunity to achieve homeostasis of the symbiont and differentiates specialized cells to harbor the symbiont [45,46]. Furthermore, they have coevolved different mechanisms to facilitate material and information exchange across the host–symbiont interface to gain more profits for both partners (Table 1).

Due to their strictly vertical transmission and the lack of genetic recombination with free-living bacteria, insect endosymbionts represent a classical coevolution with their hosts, usually causing extensive pseudogenization of the bacterial genome with subsequent loss of genes and corresponding functional pathways [86]. However, it has been shown that these obligate symbionts manage to survive for long periods in an insect host by acquiring essential genes from other co-residing microbes via horizontal gene transfer [85]. On the other hand, insects have evolved mechanisms to maintain their mutualistic relationships with endosymbionts. For example, in the *A. pisum*-*Buchnera* symbiosis, endosymbionts are embedded in a host-derived symbiosomal membrane and completely dependent on their host to meet nutritional requirements. The symbiosomal membrane is dynamic and selectively permeable, thus facilitating the bidirectional and differential movement of essential nutrients, metabolites, and biosynthetic intermediates essential to the development and survival of symbionts and the host [84].

The *R. pedestris*–*Burkholderia* symbiotic system is a typical example to explain the coevolution of defensive symbiosis. The symbiotic *Burkholderia* has a positive impact on the immunity of bean bugs, and bean bug immunity modulates the density of the symbionts [21]. *Burkholderia* extracted from bean bugs is missing the *O*-antigen, unlike cultured *Burkholderia*. Culturing *Burkholderia* for one day in yeast-glucose medium leads to restoration of the low- and high-molecular-weight *O*-antigens. *Burkholderia* undergoes drastic changes in its cell envelope during colonization in the host midgut, indicating that modification of the symbiont cell envelope is driven by the demand for the efficient communication between both partners. Modifications of the *Burkholderia* cell envelope may result in vulnerability to the host immune system and reduce its in-host survival. However, from the perspective of the host, variations in the cell envelope make it easier to control the gut symbionts. As a result, the bean bug inhibits immune responses in the symbiotic midgut region to maintain the survival of *Burkholderia* [87].

It has also been proposed that the host and beneficial heritable microbes can evolve antagonistically [88,89]. This provokes reflections about whether hosts regulate immune responses to accommodate symbionts, or if symbionts suppress host immune responses to achieve higher densities within hosts. In the aphid symbiosis, the higher load of Clade 2 *Regiella* strains exert greater survival costs on hosts than the lower load of Clade 1 strains [90]. Aphids harboring the Clade 2 *Regiella* exhibit stronger immune downregulation compared with that of the Clade 1 strain, potentially leading to variations in the load of *Regiella* strains. The immune downregulation is not a host adaptation to harbor symbionts, but rather, certain *Regiella* strains inhibit immune responses to colonize at higher loads within the host. However, some aphids have adapted to prevent immune suppression in order to control *Regiella* densities. This demonstrates that antagonistic coevolution can play a role in symbiont–insect interactions [91].

## 6. Summary and Prospect

Symbionts have shaped the evolution of their insect hosts and the symbionts themselves. Defensive symbionts particularly have a far-reaching impact on the host’s immune capacity. Symbionts can protect insects from exogenous pathogens through a variety of mechanisms, including upregulating the expression of host immune-related genes, producing antimicrobial substances, and competitively excluding pathogens. Concordantly, insects have evolved fine-tuned regulatory mechanisms to avoid overactive immune responses against symbionts or specialized cells to harbor symbionts. Alternatively, some symbionts have evolved special adaptations, such as the formation of biofilms to mediate their tolerance to host immune responses. As it has been hypothesized that symbionts could upregulate the expression of host immune genes and thereby indirectly participate in host pesticide tolerance [92], pheromone production [93], and modulation of host neuron activity [94], knowledge about colonization resistance will provide insights into the in-depth study of the contribution of symbionts to host physiology and behavior.

Knowledge about colonization resistance will also provide insights into management of insect-borne diseases and economically important insects. *Wolbachia* can reduce or even eliminate human pathogens in mosquito vectors and prevent *Flavivirus*, *Alphavirus*, *Plasmodium*, filarial parasites, and other pathogens from infecting mosquitoes. Utilization of the *Wolbachia*-mosquito association has emerged as a potential approach to control arbovirus transmission [95]. For example, introduction of the *w*Mel *Wolbachia* strain into *Ae. Aegypti* adversely influences host ability to work as a vector for *Dengue virus* [96]. In honey bees, the genetically engineered gut symbiont *Snodgrassella alvi* carrying dsRNA-producing plasmids is applied to constantly generate dsRNA in the host to target Deformed Wing Virus and *Varroa mites*, thus inducing robust protection against two parasites [97]. Although such genetic approaches are challenged with the risk of the release of genetically modified organisms, recent proof-of-concept studies demonstrate their potential and they offer novel strategies to manage insect populations [98].

Defensive symbiosis becomes a promising source for bioactive compounds with potential clinical applications. Defensive symbionts use a series of compounds from small molecules to protein toxins to help the host prevent microbial pathogens [32]. Candicidin, an effective antifungal substance against *Candida albicans*, is generated by *Streptomyces* discovered from exoskeletons of leaf-cutting ants and fungal gardens [99]. Ribosomal peptides generated by defensive symbionts may be developed into useful antimicrobials in the future. For example, mundticin KS exhibits a unique antibacterial spectrum, demonstrating potent activity only against closely related pathogens and some other bacteria. In particular, this peptide antibiotic is highly effective in treating enterococcal infection, providing insights into the battle against rapidly emerging multi-drug-resistant enterococcal pathogens in humans [100].

Despite great progress in the broad field of insect immunity, our understanding of how symbionts influence host immune responses to pathogens remains incomplete. The complexity of spatial and temporal variations among insect immune responses and the unfeasibility to culture symbionts in vitro have greatly increased the difficulty to study colonization resistance [98]. Although we now have a fundamental understanding of colonization resistance, key questions, such as the function and molecular mechanisms of the transmembrane transport of nutrients, metabolites, and biosynthetic intermediates within the host–symbiont interface [84], and the pattern by which symbionts recognize pathogens and influence the evolution of the host immune system [46] remain unanswered. Specific recognition and the molecular mechanism of PRRs also need further studies [56]. Integration of transcriptomic and metabolomic data will help to make predictions of material and information exchange between symbionts and hosts during the process of colonization resistance, which could be further verified through empirical studies [101].

## Figures and Tables

**Table 1:** Coevolution of the defensive symbiosis in insects.

Partner	Influence from Symbiosis		Selection in Evolution	Evolutionary Pattern
Insect hosts	Positive	Improved immunity	To improve communication efficiency between symbiotic partners	Symbiosomal membranes produced by the host with selective permeability of metabolites [84]
	Negative	Excessive energy consumption in immune responses	To maintain symbiont homeostasis	Degeneration of the host immune system [44] Fine-tuned regulation of immune responses by host negative regulators [45] Compartmentalization of the symbionts in specialized symbiotic organs [46]
Symbionts	Positive	Stable ecological niche (environment/nutrition)	To allocate more energetic resources to colonization resistance	Degeneration or massive pseudogenization of the genome [45,85]
	Negative	Damage from host immune responses	To avoid recognition by the host immune system and to escape from host immune responses	Formation of biofilms [79]

## Data Availability

The data presented in this study are available on request from the corresponding author.
